# How “Wound Shock” Is Cared for in France

**Published:** 1918-12

**Authors:** 


### How "Wound Shock" Is Cared for in France.

The real struggle "over there," the vital conflict, is between the opposing forces of destruction and reconstruction, between mutilation and restoration. The real antagonist of the soldier on either side is the surgeon on the opposing side, says the independent.

The surgeon has recently won a great victory. He has taken from the enemy half an army corps of his former victims. Every year some 20,000 young Britons and Frenchmen have died of shock, although their wounds were not necessarily of a fatal kind. Instead of rallying when his wounds were dressed the patient would collapse. His blood would cease to flow through the finer veins; his breathing and heart-beating would become rapid and feeble. Soon for no apparent reason he would die. No cure was known, for its cause was not known.

This was the problem that was put to the Rockefeller Institute for Medical Research and how it was solved is told in fascinating fashion by Dr. W. T. Porter in his little volume on "Shock at the Front," just published by the Atlantic Monthly. First, it was observed that death from shock was most apt to follow fractures of the femus by which the fatty marrow of the bone is brought into the blood. In the scientific process the next step after observation is experimentation. It was found that a teaspoonful of harmless olive oil injected into the veins of a cat brought on the symptoms of traumatic shock. The third step, hypothecation, led to the theory that the cause of shock was the clogging of the capillaries by minute drops of oil. The fourth and final step, the application, had to be carried on at the front and under fire where victims of femoral wounds could be operated upon without any delay.

The remedy, the best remedy so far discovered, is what to the layman would seem most absurd—impure air. By stocking the patient's head in a bag and making him breathe the air over and

